# Supplementary material for: Decomposition of retinal ganglion cell electrical images for cell type and functional inference
Source: bioRxiv. 2023 Nov 8:2023.11.06.565889. Preprint. [Version 1] doi: 10.1101/2023.11.06.565889 (PMC10659265; doi:10.1101/2023.11.06.565889)
Supplement: Supplementary file 1 [file media-1.pdf]

Supplemental Algorithms and Proofs

Eric G. Wu

1 Fitting the EI decomposition

1.1 Definitions

- The matrix $X \in \mathbb{R}^{T \times N}$ represents the EI of a neuron. This matrix contains N observed voltage waveforms, one for each of the N recording electrodes. Each observed waveform has temporal length T.
- The decomposition is parameterized by the following variables:
  - $B \in \mathbb{R}^{C \times T}$, the basis waveforms matrix, consisting of C distinct basis waveforms, each with temporal length T.
  - $A \in \mathbb{R}_{\geq 0}^{C \times N}$, the learned amplitudes (weights) on each of the basis waveforms for every recording electrode. Every element in A is required to be non-negative, because the amplitudes are interpreted as the contributions of physical compartments of the neuron to the overall recorded signal.
  - $\tau \in \mathbb{Z}^{C \times N}$, the integer-valued time shifts applied to each basis waveforms for every recording electrode.

1.2 Decomposition fitting algorithm

The EI decomposition is fitted by minimizing the mean square error between the recorded waveforms of the EI and the waveforms reconstructed from the decomposition parameters, with additional regularization terms to induce sparsity in the learned weights and to weakly constrain the compartment basis waveform shape. Let $B^{(\tau_n)}$ denote temporal shifting of each respective waveform in B by the numbers of samples specified by column n of the matrix τ, $A_{:,n}$ denote column n of the matrix A, $X_{:,n}$ denote column n of the matrix X, and $B_{:,c}$ denote row c of the matrix B. Fitting the EI decomposition corresponds to the solving the optimization problem

$$\arg\min_{B,A,\tau} \left\{ \frac{1}{2} \sum_{n=1}^{N} |B^{(\tau_n)} A_{:,n} - X_{:,n}|_2^2 + \lambda_L \sum_{i=1}^{N} r(A_{:,n}) + \frac{\lambda_p}{2} \sum_{i=1}^{C} (B_{c,:}^T - \mu_c)^T \Sigma_c^{-1} (B_{c,:}^T - \mu_c) \right\}. \quad (1)$$
$$\text{such that } A \geq 0$$

The first term in the objective, $\frac{1}{2} \sum_{n=1}^{N} |B^{(\tau_n)} A_{:,n} - X_{:,n}|_2^2$, is a least squares error term, ensuring that the decomposition accurately represents the EI. The second term, $\lambda_L \sum_{i=1}^{N} r(A_{:,n})$, is a sparsity regularizer applied to the learned weights, and has associated strength hyperparameter λ_L. $r(A_{:,n})$ is specifically chosen as an $L_{2,1}$ group-sparsity regularizer, with groups {soma, dendrite} and {axon} to allow the somatic and dendritic components to appear simultaneously on the same electrode. The final term, $\frac{\lambda_p}{2} \sum_{c=1}^{C} (B_{c,:}^T - \mu_c)^T \Sigma_c^{-1} (B_{c,:}^T - \mu_c)$, is a Gaussian prior regularizer used to control the shapes of each of the learned basis waveforms, and has associated strength hyperparameter λ_p, as well as hyperparameters μ_c and Σ_c specifying the means and covariances for each basis waveform.

The overall optimization problem (1) is not jointly convex in terms of the decomposition model parameters $\{B, A, \tau\}$. This is both because τ is integer-valued, and because of the simultaneous estimation of B and A. Due to this non-convexity, the decomposition is fitted using a novel iterative algorithm for shifted semi-non-negative matrix factorization. The algorithm alternates between two steps: (1) an amplitude and time shift optimization step, holding the compartment basis waveforms fixed; and (2) a basis waveform shape fitting step, using the previously-inferred amplitudes and time shifts to learn the waveform shape. Pseudocode for the overall fitting algorithm is provided in Algorithm 1, and mathematical details for each of the sub-steps are provided subsequently.

Algorithm 1 Alternating optimization for EI decomposition fitting

Hyperparameters: λ_L, λ_p, Σ_i, μ_i
procedure EI-DECOMPOSITION(X)
  Initialize $\mathbf{B}^{(k)}$ to the mean waveform values
  for $k \in 1, 2, \ldots, K$ **do**
    $A^{(k)}, \tau^{(k)} \leftarrow \arg\min_{A,\tau} \left\{ \frac{1}{2} \sum_{n=1}^{N} |[B^{(k)}]^{(\tau_n^{(k)})} A_{:,n} - \mathbf{x}_n|_2^2 + \lambda_L \sum_{n=1}^{N} r(A_{:,n}) \right\},\ A \geq 0$
    $B^{(k)} \leftarrow \arg\min_B \left\{ \frac{1}{2} \sum_{n=1}^{N} |B^{(\tau_n^{(k)})} A_{:,n} - \mathbf{x}_n|_2^2 + \frac{\lambda_p}{2} \sum_{c=1}^{C} (B_{c,:}^T - \mu_c)^T \Sigma_c^{-1} (B_{c,:}^T - \mu_c) \right\}$
    Normalize rows of $B^{(k)}$ to have L_2-norm 1.
  end for
  return $B, \mathbf{a}, \tau$
end procedure

1.2.1 Amplitudes and time shifts optimization

The amplitude and time shift optimization step holds the compartment basis waveforms B fixed, and uses a combination of search and convex minimization to simultaneously fit the amplitudes A and time shifts τ on every electrode. The overall objective for this step is constructed by discarding all terms that depended solely on B from the objective of problem (1), leaving the optimization problem

$$\arg\min_{A,\tau} \left\{ \frac{1}{2} \sum_{n=1}^{N} |B^{(\tau_n)} A_{:,n} - X_{:,n}|_2^2 + \lambda_L \sum_{i=1}^{N} r(A_{:,n}) \right\} \text{ such that } A \geq 0. \tag{2}$$

Though problem (2) is still not jointly convex in terms of A and τ, its structure is considerably simpler than that of the full problem (1). In particular, problem (2) is separable by electrode and can be solved separately for each electrode. In addition, if the time shifts τ are held fixed, each single-electrode problem simplifies into a regularized non-negative linear least square problem,

$$\arg\min_{A_{:,n}} \left\{ \frac{1}{2} |B^{(\tau_n)} A_{:,n} - X_{:,n}|_2^2 + \lambda_L r(A_{:,n}) \right\} \text{ such that } A_{:,n} \succeq \mathbf{0}, \tag{3}$$

which is a constrained convex minimization problem. Leveraging this property, the amplitudes and time shifts optimization is performed using a coarse-to-fine search over candidate time shifts, solving problem (3) with convex minimization for each candidate time shift, and then selecting the combination of $\{A_{:,n}, \tau_n\}$ that minimizes the objective. Because the optimization is solved separately for each recording electrode, this approach avoids combinatorial explosion over the possible combinations of time shifts. Each convex minimization problem is solved using FISTA [1], an accelerated proximal gradient descent algorithm, using a modified form of the constrained optimization formulation of the $L_{2,1}$-regularized problem described in [2] to support the additional non-negativity constraint on the learned weights. A detailed description as well as mathematical proof for this formulation is provided in Section 2.2.

Pseudocode for the amplitudes and time shifts optimization is presented in Algorithm 2. Notably, the optimization problems within both the coarse and fine searches can be easily parallelized for improved runtime.

Algorithm 2 Amplitudes and time shifts coarse-to-fine search

Hyperparameters: λ_L
procedure AMPLITUDE-TIME-SEARCH(X, B)
  for electrode $n \in 1, 2, \ldots, N$ **do**
    $\hat{V}_n \leftarrow \infty$
    $\hat{A}_{:,n}, \hat{\tau}_n \leftarrow$ empty values
    for $\tau'_n \in$ coarse grid of possible time shifts **do** ▷ Coarse search over τ
      $A'_{:,n} \leftarrow \arg\min_{\mathbf{a}_n} \left\{\frac{1}{2}|B^{(\tau_n)}A_{:,n} - X_{:,n}|_2^2 + \lambda_L r(A_{:,n})\right\}$ such that $A_{:,n} \succeq \mathbf{0}$
      $V'_n \leftarrow \frac{1}{2}|B^{(\tau'_n)}A'_{:,n} - X_{:,n}|_2^2 + \lambda_L r(A'_{:,n})$
      if $V'_n < \hat{V}_n$ **then**
        $\hat{V}_n \leftarrow V'_n$
        $\hat{A}_{:,n}, \hat{\tau}_n \leftarrow A'_{:,n}, \tau'_n$
      end if
    end for
    $V^*_n \leftarrow \infty$
    $A^*_{:,n}, \tau^*_n \leftarrow$ empty values
    for $\tau'_n in$ fine grid of time shifts in neighborhood of $\hat{\tau}_n$ **do** ▷ Fine search over τ
      $A'_{:,n} \leftarrow \arg\min_{A_{:,n}} \left\{\frac{1}{2}|B^{(\tau_n)}A_{:,n} - X_{:,n}|_2^2 + \lambda_L r(A_{:,n})\right\}$ such that $A_{:,n} \succeq \mathbf{0}$
      $V'_n \leftarrow \frac{1}{2}|B^{(\tau'_n)}A'_{:,n} - X_{:,n}|_2^2 + \lambda_L r(A'_{:,n})$
      if $V'_n < V^*_n$ **then**
        $V^*_n \leftarrow V'_n$
        $A^*_{:,n}, \tau^*_n \leftarrow \mathbf{a}'_n, \tau'_n$
      end if
    end for
  end for
  return A^*, T^*
end procedure

1.2.2 Compartment basis waveform shape optimization

The compartment basis waveform shape optimization step holds the amplitudes A and time shifts τ fixed, and solves the convex quadratic minimization problem

$$\arg\min_{B} \left\{ \frac{1}{2}\sum_{n=1}^{N} |B^{(\tau_n)}\mathbf{a}_n - \mathbf{x}_n|_2^2 + \frac{\lambda_P}{2}\sum_{c=1}^{C}(\mathbf{b}_c - \mu_c)^T \Sigma_C^{-1}(\mathbf{b}_c - \mu_c)\right\} \tag{4}$$

for the basis waveform shapes B. Construction of the matrix $B^{(\tau_n)}$ requires temporal shifting of the basis waveforms comprising B, complicating computer representations of problem (4). Because a circular shift of t samples in time domain can be expressed simply in Fourier domain as multiplication by the complex phase shift $e^{-i\omega t}$, the difficulty of representing time shifts is alleviated by solving the problem in Fourier domain. Letting $\tilde{B}$ denote the discrete Fourier transform of the basis waveforms, $\tilde{X}$ the discrete Fourier transform of the observed EI, $\odot$ Hadamard (element-wise) matrix multiplication, $\tilde{P}^{(\tau_n)}$ the complex-valued Fourier phase shift matrix corresponding to temporally shifting the compartment basis waveforms by the amounts specified in τ_n, and G the stacked real-imaginary discrete Fourier transform analysis matrix (see Section 3.2 for definition), problem (4) is expressed in Fourier domain as

$$\arg\min_{\tilde{B}} \left\{ \frac{1}{2}\sum_{i=1}^{N} |(\tilde{B} \odot \tilde{P}^{(\tau_n)})A_{:,n} - \tilde{X}_{:,n}|_2^2 + \frac{\lambda_P}{2}\sum_{c=1}^{C}(GB_{c,:}^T - G\mu_c)^T (G\Sigma_c G)^{-1}(GB_{c,:}^T - G\mu_c)\right\}. \tag{5}$$

This formulation is equivalent to the original time domain problem (4) due to Parseval's relation. As problem (5) corresponds to minimization of a convex quadratic, it is solved by constructing and solving a system of linear equations derived from the first-order conditions of optimality. A complete derivation of the coefficients of this linear system is provided in Section 3.

2 Non-negative $L_{2,1}$-regularized minimization

The EI decomposition fitting procedure solves problem (3) as a non-negative $L_{2,1}$-regularized linear least squares problem. Because the $L_{2,1}$ regularizer is not smooth, this problem cannot be solved directly with gradient descent. Following [2], we reformulate the problem into a different constrained convex minimization problem that can be easily solved using proximal gradient descent. The derivation of the proximal operator for the re-formulated problem builds heavily on the original derivation of the unconstrained $L_{2,1}$-regularized proximal operator performed in [2], and so we repeat that derivation first.

2.1 Unconstrained $L_{2,1}$-regularized minimization

The derivation for the proximal algorithm solving the unconstrained $L_{2,1}$-regularized convex minimization problem (e.g. without the non-negativity constraint) is taken from [2].

Consider the *unconstrained* $L_{2,1}$-regularized convex minimization problem

$$\min_{W} \left\{ L(W) + \rho |W|_{2,1} \right\} \tag{6}$$

where $L(W)$ is a smooth convex loss function in the variable W. This problem cannot be solved using vanilla gradient descent, because the $L_{2,1}$ penalty term $|W|_{2,1}$ is not differentiable everywhere (e.g. at the origin). Reference [2] replaces this unconstrained non-smooth problem with the constrained smooth optimization problem

$$\min_{(W,\mathbf{t})\in D} \left\{ L(W) + \rho \sum_{i=1}^{G} t_i \right\} \tag{7}$$

where D is the convex set $D = \{(W, \mathbf{t}) \mid |\mathbf{w}_i|_2 \leq t_i,\ i \in 1, 2, ..., G\}$ and G is the number of groups in the $L_{2,1}$ group-sparsity regularizer. By inspection, D is convex, since it is the Cartesian product of ice-cream-cones. The objective in problem (7) is smooth and convex, and thus problem (7) is a constrained smooth convex minimization problem. This problem can be solved using proximal gradient descent or one of its accelerated variants (e.g. FISTA [1]), provided that projection onto the convex set D can be performed efficiently. This projection problem, projecting an arbitrary $(U, \mathbf{v})$ onto the convex set D, is expressed as

$$\pi_D(W, \mathbf{t}) = \arg \min_{(U,\mathbf{v})\in D} \left\{ \frac{1}{2}|W - U|_F^2 + \frac{1}{2}|\mathbf{t} - \mathbf{v}|_2^2 \right\}. \tag{8}$$

Equation (8) can be separated by group, resulting in

$$\pi_D(W, \mathbf{t}) = \arg \min_{(U,\mathbf{v})\in D} \left\{ \sum_{i=1}^{G} \left[\frac{1}{2}|\mathbf{w}_i - \mathbf{u}_i|^2 + \frac{1}{2}(t_i - v_i)^2 \right] \right\}. \tag{9}$$

Assuming that none of the variables in W are shared between groups, problem (9) is solved found by separately solving each of the one-group problems

$$\pi_{D_i}(\mathbf{w}_i, t_i) = \arg \min_{(\mathbf{u}_i,v_i)\in D} \left\{ \frac{1}{2}|\mathbf{w}_i - \mathbf{u}_i|^2 + \frac{1}{2}(t_i - v_i)^2 \right\}. \tag{10}$$

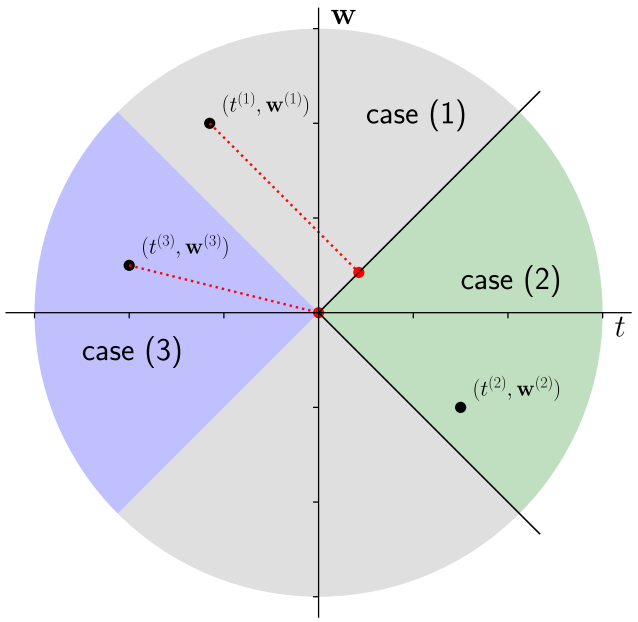

Figure 1: Projection onto the 1D ice-cream cone. There are three possible cases: (1) $|w_i| > |t_i|$, corresponding to the gray shaded region; (2) $|w_i| \leq |t_i|$, corresponding to the green shaded feasible region; (3) $|w_i| \leq -t_i$, corresponding to the blue shaded region. In cases (1) and (3), (w_i, t_i) lies outside the feasible region, and the associated dotted red lines and points correspond to the minimum norm projections onto the feasible set.

Consider the case where $\mathbf{w}_i$ is one-dimensional. A drawing of the corresponding two-dimensional $(\mathbf{w}_i, t_i)$ coordinate space illustrating the three possible cases for projection is provided in Figure 1. The first case, $|w| > |t|$, corresponds to the gray shaded region of Figure 1. This is outside the feasible region, and the projection corresponds the orthogonal projection onto the edge of the ice-cream cone. The second case, $|w| \leq |t|$, corresponds to the green shaded region of Figure 1. This is already feasible, so nothing needs to be done. The third case, $|w| \leq -t$, corresponds to the blue shaded region of Figure 1. This is outside the feasible region, and the solution is to project onto the origin. Formally, these rules can be expressed as

$$u_i^* = \begin{cases} \frac{w_i + t_i}{2}, \ |w_i| > |t_i| & \text{(case 1)} \\ w_i, \ |w_i| \leq t_i & \text{(case 2)} \\ 0, \ |w_i| \leq -t_i & \text{(case 3)} \end{cases}$$

$$v_i^* = \begin{cases} \frac{w_i + t_i}{2}, \ |w_i| > |t_i| & \text{(case 1)} \\ v_i, \ |w_i| \leq t_i & \text{(case 2)} \\ 0, \ |w_i| \leq -t_i & \text{(case 3)} \end{cases}$$

Because the cone is rotationally-symmetric about the t axis, the solution to the one-dimensional projection problem can be generalized to N dimensions as

$$\mathbf{u}_i^* = \begin{cases} \frac{|\mathbf{w}_i|+t_i}{2}\frac{\mathbf{w}_i}{|\mathbf{w}_i|}, \ |\mathbf{w}_i| > |t_i| & \text{(case 1)} \\ \mathbf{w}_i, \ |\mathbf{w}_i| \le t_i & \text{(case 2)} \\ \mathbf{0}, \ |\mathbf{w}_i| \le -t_i & \text{(case 3)} \end{cases} \tag{11}$$

$$t_i^* = \begin{cases} \frac{|\mathbf{w}_i|+t_i}{2}, \ |\mathbf{w}_i| > |t_i| & \text{(case 1)} \\ t_i, \ |\mathbf{w}_i| \le t_i & \text{(case 2)} \\ 0, \ |\mathbf{w}_i| \le -t_i & \text{(case 3)} \end{cases} \tag{12}$$

Proof. (11) and (12) solve the optimization problem (10).

The proof is performed by verifying that (11) and (12) satisfy the following condition of optimality for constrained smooth convex minimization problems: *For the convex minimization problem* $\arg\min_{\mathbf{x}\in F}\{L(\mathbf{x})\}$ *with smooth convex objective* $L(\mathbf{x})$ *and convex feasible set* F*, the point* $\mathbf{x}^* \in F$ *is optimal if and only if* $\langle \mathbf{x} - \mathbf{x}^*, L'(\mathbf{x}^*)\rangle \geq 0$*,* $\forall \mathbf{x} \in F$. The gradient of the objective in (10) with respect to $(\mathbf{u}_i, v_i)$ is $(\mathbf{u}_i - \mathbf{w}_i, v_i - t_i)$. Consider each of the three possible cases:

- Case (1), $|\mathbf{w}_i| > t_i$. Define $\alpha = \frac{t_i - |\mathbf{w}_i|}{2}\frac{\mathbf{w}_i}{|\mathbf{w}_i|}$ and $\beta = \frac{|w_i| - t_i}{2}$. By inspection, $|\alpha| = \beta > 0$, where the inequality is satisfied due to the condition.

  Plugging this into the left hand side of the optimality condition, we get

$$\begin{aligned} \langle \mathbf{u}_i - \mathbf{u}_i^*, \mathbf{u_i}^* - \mathbf{w}_i\rangle + (v_i - v_i^*)(v_i^* - t_i) &= \langle \mathbf{u}_i, \alpha\rangle + t_i\beta \\ &\geq -|\mathbf{u}_i||\alpha| + t_i\beta \\ &\geq -t_i\beta + t_i\beta \\ &\geq 0 \end{aligned}$$

  where the first inequality is due to Cauchy-Schwarz, and the second inequality is due to $|\mathbf{u}_i| < v_i$, which results from the constraint $(\mathbf{u}_i, v_i) \in D$. Thus case (1) of (11) and (12) is proven.

- Case (2), $|\mathbf{w}_i| \leq t_i$. In this case, $(\mathbf{u}_i^*, v_i^*) = (\mathbf{w}_i, t_i)$ is in the feasible set and minimizes the single-group objective function (10). Thus case (2) of (11) and (12) is proven.

- Case (3), $|\mathbf{w}_i| \leq -t_i$. we write

$$\begin{aligned} \langle \mathbf{u}_i - \mathbf{u}_i^*, \mathbf{u_i}^* - \mathbf{w}_i\rangle + (v_i - v_i^*)(v_i^* - t_i) &= \langle \mathbf{u}_i, -\mathbf{w}_i\rangle + (v_i)(-t_i) \\ &\geq -|\mathbf{u}_i||\mathbf{w}_i| - t_i v_i \\ &\geq -t_i(-v_i) - t_i v_i \\ &\geq 0 \end{aligned}$$

  where the first inequality is due to the Cauchy-Schwarz inequality, and the second inequality comes from the conditions $|\mathbf{w}_i| \leq -t_i$ and $t_i \leq 0$. Thus case (3) of equations (11) and (12) is proven.

Thus the claim is proven. □

The overall FISTA algorithm solving the unconstrained $L_{2,1}$-regularized problem as a constrained convex minimization problem is given in Algorithm 3. Note that $f_{\gamma,W_i,\mathbf{t}_i}(W_{i+1}, \mathbf{t}_{i+1})$ in the back-tracking line search is defined as

$$\begin{aligned} f_{\gamma,W_i,\mathbf{t}_i}(W, \mathbf{t}) \triangleq f(W_i, \mathbf{t}_i) + \langle \boldsymbol{\nabla}'_W f(W', \mathbf{t}')|_{W'=W_i,\mathbf{t}'=\mathbf{t}_i}, W - W_i\rangle + \\ \langle \boldsymbol{\nabla}_{\mathbf{t}'} f(W', \mathbf{t}')|_{W'=W_i,\mathbf{t}'=\mathbf{t}_i}, \mathbf{t} - \mathbf{t}_i\rangle + \frac{\gamma}{2}|W - W_i|_F^2 + \frac{\gamma}{2}|\mathbf{t} - \mathbf{t}_i|_2^2 \end{aligned} \tag{13}$$

and π_{D_G} corresponds to the projection operator from equation (8).

Algorithm 3 FISTA for unconstrained $L_{2,1}$-regularized problem

Hyperparameter ρ_{L21}
procedure UNCONSTRAINED-FISTA-L21($L(W)$, W_0, G, γ)
  Randomly initialize variable $\mathbf{t} \in \mathbb{R}_{\geq 0}^{|G|}$
  $f(W, \mathbf{t}) \triangleq L(W) + \rho_{L21} \mathbf{1}^T \mathbf{t}$
  Initialize $i = 1, W_1 = W_0, b_{-1} = 0, b_0 = 1$
  $W^\star = W_0$
  repeat
    $\alpha_i = \frac{b_{i-2} - 1}{b_{i-1}}$
    $W_i' = W_i + \alpha_i (W_i - W_{i-1})$
    $\mathbf{t}_i' = \mathbf{t}_i + \alpha_i (\mathbf{t}_i - \mathbf{t}_{i-1})$
    while True **do** ▷ Backtracking line search
      $\gamma = \frac{\gamma}{2}$
      $\bar{W}_{i+1} = W_i' - \frac{1}{\gamma} \nabla_W f(W, \mathbf{t})\big|_{W=W_i', \mathbf{t}=\mathbf{t}_i'}$
      $\bar{\mathbf{t}}_{i+1} = \mathbf{t}_i' - \frac{1}{\gamma} \nabla_{\mathbf{t}} f(W, \mathbf{t})\big|_{W=W_i', \mathbf{t}=\mathbf{t}_i'}$
      $W_{i+1}, \mathbf{t}_{i+1} = \pi_{D_G}(\bar{W}_{i+1}, \bar{\mathbf{t}}_{i+1})$
      if $f(W_{i+1}, \mathbf{t}_{i+1}) \leq f_{\gamma, W_i, \mathbf{t}_i}(W_{i+1}, \mathbf{t}_{i+1})$ **then**
        $W^\star = W_{i+1}$
        break
      end if
    end while
    $b_i = \frac{1 + \sqrt{1 + 4b_{i-1}^2}}{2}$
  until Converged
  return $W^\star$
end procedure

2.2 $L_{2,1}$-regularized minimization with non-negativity constraint

This derivation is performed by the authors.

For the EI decomposition, the learned compartment amplitudes are required to be non-negative, corresponding to the constrained minimization problem

$$\arg\min_{W \geq 0} \left\{ L(W) + \rho |W|_{2,1} \right\} \tag{14}$$

where $W \geq 0$ constrains all variables in W to be non-negative, $L(W)$ is a smooth convex function of W, and the $L_{2,1}$ penalty $|W|_{2,1}$ is not smooth. Like before, problem (14) is reformulated as a smooth constrained optimization problem

$$\arg\min_{(W, \mathbf{t}) \in C} \left\{ L(W) + \rho \sum_{i=1}^{G} t_i \right\} \tag{15}$$

where C is the set $C = \{(W, \mathbf{t}) \mid \mathbf{w}_i \geq \mathbf{0}, |\mathbf{w}_i|_2 \leq t_i,\ i \in 1, 2, ..., G\}$, and G is the number of groups in the group-sparsity regularizer. C is a convex set, since it is the Cartesian product of intersections of convex sets (in particular, an ice-cream cone and the non-negative orthant), which must be convex.

The projection onto the convex set C is given by the optimization problem

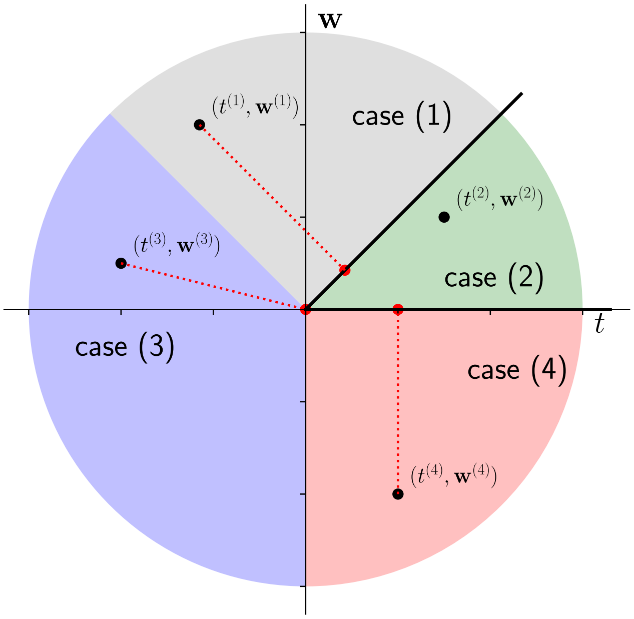

Figure 2: Projection onto the 1D non-negative subset of the ice-cream cone. There are now four cases: (1) $w_i \geq |t_i|$, corresponding to the gray shaded region; (2) $w_i \geq 0, w_i \leq t_i$, corresponding to the green shaded region; (3) $t_i < 0, w_i < -t_i$, corresponding to the blue shaded region; and (4) $w_i < 0, t_i > 0$, corresponding to the red shaded region. In cases (1), (3), and (4), (w_i, t_i) lies outside the feasible region, and the associated red dotted lines and points correspond to the minimum norm projections onto the feasible set.

$$\pi_C(W, \mathbf{t}) = \arg \min_{(U,\mathbf{v})\in C} \left\{ \frac{1}{2}|W - U|_F^2 + \frac{1}{2}|\mathbf{t} - \mathbf{v}|_2^2 \right\}. \tag{16}$$

As before, the objective in (16) is separated by group, yielding

$$\pi_C(W, \mathbf{t}) = \arg \min_{(U,\mathbf{v})\in C} \left\{ \sum_{i=1}^{G} \left[\frac{1}{2}|\mathbf{w}_i - \mathbf{u}_i|^2 + \frac{1}{2}(t_i - v_i)^2 \right] \right\}. \tag{17}$$

Assuming that no variables are shared between groups, problem (17) can be solved separately for each group, yielding

$$\pi_{C_i}(\mathbf{w}_i, t_i) = \arg \min_{(\mathbf{u}_i,v_i)\in C} \left\{ \frac{1}{2}|\mathbf{w}_i - \mathbf{u}_i|^2 + \frac{1}{2}(t_i - v_i)^2 \right\}. \tag{18}$$

Consider the case where $\mathbf{w}_i$ is one-dimensional. A drawing of the two-dimensional $(\mathbf{w}_i, t_i)$ coordinate space is provided in Figure 2 to illustrate the four possible cases for projection:

1. $w_i \geq |t_i|$, corresponding to the gray shaded region of Figure 2. In this case, the solution is to orthogonally project (w_i, t_i) onto the $w_i = v_i$ line.

2. $w_i \geq 0, w_i \leq t_i$, corresponding to the green shaded region of Figure 2. In this case, the point (w_i, t_i) is already feasible, and so nothing needs to be done.

3. $t_i < 0, w_i < -t_i$, corresponding to the blue shaded region of Figure 2. In this case, the projection is performed by mapping (w_i, t_i) onto the origin.

4. $w_i < 0, t_i > 0$, corresponding to the red shaded region of Figure 2. In this case, the projection is performed by mapping (w_i, t_i) to $(0, t_i)$.

In mathematical terms, this corresponds to

$$u_i^* = \begin{cases} \frac{w_i+t_i}{2},\ w_i \geq |t_i| & \text{(case 1)} \\ w_i,\ w_i \geq 0, w_i \leq t_i & \text{(case 2)} \\ 0,\ t_i < 0, w_i < -t_i & \text{(case 3)} \\ 0,\ w_i < 0, t_i > 0 & \text{(case 4)} \end{cases} \tag{19}$$

and

$$v_i^* = \begin{cases} \frac{w_i+t_i}{2},\ w_i \geq |t_i| & \text{(case 1)} \\ t_i,\ w_i \geq 0, w_i \leq t_i & \text{(case 2)} \\ 0,\ t_i < 0, w_i < -t_i & \text{(case 3)} \\ t_i,\ w_i < 0, t_i > 0 & \text{(case 4).} \end{cases} \tag{20}$$

Unlike the unconstrained $L_{2,1}$ case, the projection is no longer rotationally symmetric about the t_i-axis because of the non-negativity constraint. Thus, generalizing the above to the N-dimensional case requires accounting for the possibility that some but not all of the components of $\mathbf{w}_i$ lie outside the non-negative quadrant.

The situation where some but not all of the components of $\mathbf{w}_i$ lie outside the non-negative quadrant is now examined in greater detail. Define $J_i^{(+)} = \{j \in 1, 2, \ldots, N \mid w_i^{(j)} \geq 0\}$ (e.g. the set of indices j where $w_i^{(j)} \geq 0$), and define $J_i^{(-)} = \{j \in 1, 2, ..., N \mid w_i^{(j)} < 0\}$ (e.g. the set of indices j where $w_i^{(j)} < 0$). All indices must belong to exactly one of $J_i^{(+)}$ or $J_i^{(-)}$. The single-group objective function from (18) is then split into

$$\frac{1}{2}|\mathbf{w}_i - \mathbf{u}_i|^2 + \frac{1}{2}(t_i - v_i)^2 = \frac{1}{2}\sum_{j \in J_i^{(+)}} [\mathbf{w}_i^{(j)} - \mathbf{u}_i^{(j)}]^2 + \frac{1}{2}\sum_{j \in J_i^{(-)}} [\mathbf{w}_i^{(j)} - \mathbf{u}_i^{(j)}]^2 + \frac{1}{2}(t_i - v_i). \tag{21}$$

First, consider only the variables $\{\mathbf{u}_i^{(j)} \mid j \in J_i^{(-)}\}$ (corresponding to the negative-valued components of $\mathbf{w}_i$ only). These variables are included only in the first term of (21). No matter the value of t_i, the optimal value for each of these variables $\{(\mathbf{u}_i^{(j)})^* \mid j \in J_i^{(-)}\}$ is exactly 0, corresponding to the lower $w < 0$ half of Figure 2. Intuitively, this is because all nonzero feasible values of $\{\mathbf{u}_i^{(j)} \mid j \in J_i^{(-)}\}$ are positive, and thus would increase the value of $[\mathbf{w}_i^{(j)} - \mathbf{u}_i^{(j)}]^2$.

Then, consider the remaining variables in the problem, v_i and $\{\mathbf{u}_i^{(j)} \mid j \in J_i^{(+)}\}$. These variables lie in the upper $w \geq 0$ half of Figure 2, and thus, for these particular variables, the projection operator $\pi_D(\mathbf{w}, t)$ previously developed in Section 2.1 for the unconstrained regularized problem can be applied to solve for v_i and $\{\mathbf{u}_i^{(j)} \mid j \in J_i^{(+)}\}$.

Letting max denote the element-wise maximum operator, the projection operator for the non-negative regularized problem can be expressed concisely as

$$\pi_C(\mathbf{w}_i, t_i) = \pi_D(\max(\mathbf{w}_i, \mathbf{0}), t_i). \tag{22}$$

The overall FISTA algorithm solving the non-negative $L_{2,1}$-regularized problem is given in Algorithm 4. Like before, $f_{\gamma, W_i, \mathbf{t}_i}(W_{i+1}, \mathbf{t}_{i+1})$ in the backtracking line search is defined as

$$f_{\gamma, W_i, \mathbf{t}_i}(W, \mathbf{t}) \triangleq f(W_i, \mathbf{t}_i) + \langle \boldsymbol{\nabla}'_W f(W', \mathbf{t}')|_{W'=W_i, \mathbf{t}'=\mathbf{t}_i}, W - W_i \rangle + \\ \langle \boldsymbol{\nabla}_{\mathbf{t}'} f(W', \mathbf{t}')|_{W'=W_i, \mathbf{t}'=\mathbf{t}_i}, \mathbf{t} - \mathbf{t}_i \rangle + \frac{\gamma}{2} |W - W_i|_F^2 + \frac{\gamma}{2} |\mathbf{t} - \mathbf{t}_i|_2^2.$$

Algorithm 4 FISTA for non-negative $L_{2,1}$-regularized problem

Hyperparameter ρ_{L21}
procedure NonNegative-FISTA-L21($L(W)$, W_0, G, γ)
    Randomly initialize variable $\mathbf{t} \in \mathbb{R}^{|G|}_{\geq 0}$
    $f(W, \mathbf{t}) \triangleq L(W) + \rho_{L21} \mathbf{1}^T \mathbf{t}$
    Initialize $i = 1, W_1 = W_0, b_{-1} = 0, b_0 = 1$
    $W^\star = W_0$
    repeat
        $\alpha_i = \frac{b_{i-2} - 1}{b_{i-1}}$
        $W'_i = W_i + \alpha_i (W_i - W_{i-1})$
        $\mathbf{t}'_i = \mathbf{t}_i + \alpha_i (\mathbf{t}_i - \mathbf{t}_{i-1})$
        while True **do** ▷ Backtracking line search
            $\gamma = \frac{\gamma}{2}$
            $\bar{W}_{i+1} = W'_i - \frac{1}{\gamma} \boldsymbol{\nabla}_W f(W, \mathbf{t})\big|_{W=W'_i, \mathbf{t}=\mathbf{t}'_i}$
            $\bar{\mathbf{t}}_{i+1} = \mathbf{t}'_i - \frac{1}{\gamma} \boldsymbol{\nabla}_{\mathbf{t}} f(W, \mathbf{t})\big|_{W=W'_i, \mathbf{t}=\mathbf{t}'_i}$
            $W_{i+1}, \mathbf{t}_{i+1} = \pi_{D_G}(\max(\bar{W}_{i+1}, 0), \bar{\mathbf{t}}_{i+1})$
            if $f(W_{i+1}, \mathbf{t}_{i+1}) \leq f_{\gamma, W_i, \mathbf{t}_i}(W_{i+1}, \mathbf{t}_{i+1})$ **then**
                $W^\star = W_{i+1}$
                break
            end if
        end while
        $b_i = \frac{1 + \sqrt{1 + 4 b_{i-1}^2}}{2}$
    until Converged
    return $W^\star$
end procedure

3 Solving waveform shapes linear system in Fourier domain

This section describes the construction of the linear system of equations associated with the first-order conditions of optimality for the waveform shapes fitting step. This optimization is performed in the Fourier domain, to simplify accounting for the basis waveform temporal shifts, and thus the coefficients of the linear system are found by differentiating the mean square error loss function with respect to the real and imaginary components of the basis waveforms.

The construction of the linear system is rather tedious; for simplicity we start by first finding the linear coefficients for the unregularized waveform shapes problem (without the Gaussian shape prior), and then derive the modifications needed to solve the regularized waveform shapes problem (with the Gaussian shape prior).

3.1 Solving for basis waveforms without the Gaussian shape prior

Let $\tilde{B}$ denote the discrete Fourier transform (DFT) of the basis waveforms B, $\tilde{X}_{:,l}$ the DFT of the lth observed data waveform, $\tilde{P}_l$ the complex-valued Fourier phase shift matrix corresponding to the previously-estimated time shifts for the lth data waveform, $A_{:,l}$ the previously-estimated amplitudes for the lth data waveform, and $\odot$ Hadamard element-wise matrix multiplication. The least squares optimization problem in frequency domain over all observed data waveforms for a given cell is

$$\arg\min_{\tilde{B}} \left\{ \sum_{l=1}^{L} \frac{1}{2} |(\tilde{B} \odot \tilde{P^{(l)}})A_{:,l} - \tilde{X}_{:,l}|_2^2 \right\}. \tag{23}$$

This is an unconstrained convex minimization problem, and has first order condition of optimality

$$\nabla_{\tilde{B}} \left\{ \sum_{l=1}^{L} \frac{1}{2} |(\tilde{B} \odot \tilde{P^{(l)}} A_{:,l} - \tilde{X}_{:,l}|_2^2 \right\} = 0. \tag{24}$$

Interchanging summation and the gradient operator leaves

$$\sum_{l=1}^{L} \nabla_{\tilde{B}} \left\{ \frac{1}{2} |(\tilde{B} \odot \tilde{P^{(l)}})A_{:,l} - \tilde{X}_{:,l}|_2^2 \right\} = 0. \tag{25}$$

Let the subscript Re correspond to the real component of a complex number (e.g. $\tilde{y}_{\text{Re}} = \text{Re}\{\tilde{y}\}$), and let the subscript Im correspond to the imaginary component of a complex number (e.g. $\tilde{y}_{\text{Im}} = \text{Im}\{\tilde{y}\}$). Furthermore, define $\mathscr{L}_l \triangleq \frac{1}{2}|(\tilde{B} \odot \tilde{P^{(l)}})A_{:,l} - \tilde{X}_{:,l}|_2^2$ as the contribution of data waveform l to the overall mean square error. Taking the derivative of $\mathscr{L}_l$ with respect to $(\tilde{B}_{\text{Re}})_{f,k}$, the real component of the fth Fourier coefficient of the kth basis waveform, results in

$$\begin{aligned}
\frac{\partial \mathscr{L}_l}{\partial (\tilde{B}_{\text{Re}})_{f,k}} = A_{k,l} \sum_j & \left\{ (\tilde{B}_{\text{Re}})_{f,j} A_{j,l} [(\tilde{P}^{(l)}_{\text{Re}})_{f,k} (\tilde{P}^{(l)}_{\text{Re}})_{f,j} + (\tilde{P}^{(l)}_{\text{Im}})_{f,k} (\tilde{P}^{(l)}_{\text{Im}})_{f,j}] \right\} \\
+ A_{k,l} \sum_j & \left\{ (\tilde{B}_{\text{Im}})_{f,j} A_{j,l} [-(\tilde{P}^{(l)}_{\text{Re}})_{f,k} (\tilde{P}^{(l)}_{\text{Im}})_{f,j} + (\tilde{P}^{(l)}_{\text{Im}})_{f,k} (\tilde{P}^{(l)}_{\text{Re}})_{f,j}] \right\} \\
& - (\tilde{P}^{(l)}_{\text{Re}})_{f,k} (\tilde{X}_{\text{Re}})_{f,l} A_{k,l} - (\tilde{P}^{(l)}_{\text{Im}})_{f,k} (\tilde{X}_{\text{Im}})_{f,l} A_{k,l}.
\end{aligned} \tag{26}$$

Similarly, taking the derivative of $\mathscr{L}_l$ with respect to $(\tilde{B}_{\text{Im}})_{f,k}$, the imaginary component of the fth Fourier coefficient of the kth basis waveform, results in

$$\frac{\partial \mathscr{L}_l}{\partial (\tilde{B}_{\mathrm{Im}})_{f,k}} = A_{k,l} \sum_j \left\{ (\tilde{B}_{\mathrm{Re}})_{f,j} A_{j,l} [(\tilde{P}^{(l)}_{\mathrm{Re}})_{f,k} (\tilde{P}^{(l)}_{\mathrm{Im}})_{f,j} - (\tilde{P}^{(l)}_{\mathrm{Im}})_{f,k} (\tilde{P}^{(l)}_{\mathrm{Re}})_{f,j}] \right\}$$
$$+ A_{k,l} \sum_j \left\{ (\tilde{B}_{\mathrm{Im}})_{f,j} A_{j,l} [(\tilde{P}^{(l)}_{\mathrm{Im}})_{f,k} (\tilde{P}^{(l)}_{\mathrm{Im}})_{f,j} + (\tilde{P}^{(l)}_{\mathrm{Re}})_{f,k} (\tilde{P}^{(l)}_{\mathrm{Re}})_{f,j}] \right\}$$
$$- (\tilde{P}^{(l)}_{\mathrm{Re}})_{f,k} (\tilde{X}_{\mathrm{Im}})_{f,l} A_{k,l} + (\tilde{P}^{(l)}_{\mathrm{Im}})_{f,k} (\tilde{X}_{\mathrm{Re}})_{f,l} A_{k,l}. \tag{27}$$

3.1.1 Constructing linear equations from the first-order conditions

The optimal waveform shapes are found by solving a system of linear equations in terms of $(\tilde{B}_{\mathrm{Re}})_{f,k}$ and $(\tilde{B}_{\mathrm{Im}})_{f,k}$ corresponding to the first-order conditions of optimality. Two families of equations are constructed, the first by differentiating with respect to the $\tilde{B}_{\mathrm{Re}}$, and the second by differentiating with respect to $\tilde{B}_{\mathrm{Im}}$.

First equation family: differentiating with respect to $\tilde{B}_{\mathrm{Re}}$ Consider the first-order condition associated with differentiating the objective with respect to $(\tilde{B}_{\mathrm{Re}})_{f,k}$, the real component of the fth Fourier coefficient of the kth basis waveform:

$$\sum_{l=1}^{L} \frac{\partial}{\partial (\tilde{B}_{\mathrm{Re}})_{f,k}} \left\{ \frac{1}{2} |(\tilde{B} \odot \tilde{P^{(l)}}) A_{:,l} - \tilde{X}_{:,l}|_2^2 \right\} = \sum_{l=1}^{L} \frac{\partial \mathscr{L}_l}{\partial (\tilde{B}_{\mathrm{Re}})_{f,k}} = 0. \tag{28}$$

$(\tilde{B}_{\mathrm{Re}})_{f,k}$ and $(\tilde{B}_{\mathrm{Im}})_{f,k}$ for each of the basis waveforms $k \in \{1, 2, ..., K\}$ appear in this equation, and hence there are $2K$ unknowns in this equation. Let $[(\tilde{B}_{\mathrm{Re}})_{f,k}]^{(\mathrm{Re},f,k)}$ denote the coefficient of $(\tilde{B}_{\mathrm{Re}})_{f,k}$, and $[(\tilde{B}_{\mathrm{Im}})_{f,k}]^{(\mathrm{Re},f,k)}$ denote the coefficient of $(\tilde{B}_{\mathrm{Im}})_{f,k}$ in this equation, where the superscript (Re, f, k) serves as a reminder that the equation was constructed by differentiating with respect to the real component of the fth Fourier coefficient of the kth basis waveform. The coefficients of the equation are

$$[(\tilde{B}_{\mathrm{Re}})_{f,k}]^{(\mathrm{Re},f,k)} = \sum_{l=1}^{L} \left\{ A_{k,l} A_{j,l} [(\tilde{P}^{(l)}_{\mathrm{Re}})_{f,k} (\tilde{P}^{(l)}_{\mathrm{Re}})_{f,j} + (\tilde{P}^{(l)}_{\mathrm{Im}})_{f,k} (\tilde{P}^{(l)}_{\mathrm{Im}})_{f,j}] \right\} \tag{29}$$

and

$$[(\tilde{B}_{\mathrm{Im}})_{f,k}]^{(\mathrm{Re},f,k)} = \sum_{l=1}^{L} \left\{ A_{k,l} A_{j,l} [(\tilde{P}^{(l)}_{\mathrm{Im}})_{f,k} (\tilde{P}^{(l)}_{\mathrm{Re}})_{f,j} - (\tilde{P}^{(l)}_{\mathrm{Re}})_{f,k} (\tilde{P}^{(l)}_{\mathrm{Im}})_{f,j}] \right\} \tag{30}$$

The right hand side of the equation $R^{(\mathrm{Re},f,k)}$ is constructed from the third terms of each of equations (26) and (27):

$$R^{(\mathrm{Re},f,k)} = \sum_{l=1}^{L} \left\{ (\tilde{P}^{(l)}_{\mathrm{Re}})_{f,k} (\tilde{X_{\mathrm{Re}}})_{f,l} A_{k,l} + (\tilde{P}^{(l)}_{\mathrm{Im}})_{f,k} (\tilde{X_{\mathrm{Im}}})_{f,l} A_{k,l} \right\} \tag{31}$$

Thus, the overall linear equation associated with the derivative with respect to $(\tilde{B}_{\mathrm{Re}})_{f,k}$ is

$$\sum_{j=1}^{K} [(\tilde{B}_{\mathrm{Re}})_{f,j}]^{(\mathrm{Re},f,k)} (\tilde{B}_{\mathrm{Re}})_{f,j} + \sum_{j=1}^{K} [(\tilde{B}_{\mathrm{Im}})_{f,j}]^{(\mathrm{Re},f,k)} (\tilde{B}_{\mathrm{Im}})_{f,j} = R^{(\mathrm{Re},f,k)}. \tag{32}$$

Second equation family: differentiating with respect to $\tilde{B}_{\mathrm{Im}}$ Consider the first-order condition associated with differentiating the objective with respect to $(\tilde{B}_{\mathrm{Im}})_{f,k}$, the imaginary component of the fth Fourier coefficient of the kth basis waveform:

$$\sum_{l=1}^{L} \frac{\partial}{\partial(\tilde{B}_{\mathrm{Im}})_{f,k}} \left\{ \frac{1}{2} |(\tilde{B} \odot \tilde{P^{(l)}}) A_{:,l} - \tilde{X}_{:,l}|_2^2 \right\} = \sum_{l=1}^{L} \frac{\partial \mathscr{L}_l}{\partial(\tilde{B}_{\mathrm{Im}})_{f,k}} = 0. \tag{33}$$

$(\tilde{B}_{\mathrm{Re}})_{f,k}$ and $(\tilde{B}_{\mathrm{Im}})_{f,k}$ for each of the basis waveforms $k \in \{1, 2, ..., K\}$ appear in this equation, and hence there are $2K$ unknowns in this equation. Let $[(\tilde{B}_{\mathrm{Re}})_{f,k}]^{(\mathrm{Im},f,k)}$ denote the coefficient of $(\tilde{B}_{\mathrm{Re}})_{f,k}$, and $[(\tilde{B}_{\mathrm{Im}})_{f,k}]^{(\mathrm{Im},f,k)}$ denote the coefficient of $(\tilde{B}_{\mathrm{Im}})_{f,k}$ in this equation, where the superscript (Im, f, k) serves as a reminder that the equation was constructed by differentiating with respect to the imaginary component of the fth Fourier coefficient of the kth basis waveform. The coefficients of the equation are

$$[(\tilde{B}_{\mathrm{Re}})_{f,k}]^{(\mathrm{Im},f,k)} = \sum_{l=1}^{L} \{A_{k,l} A_{j,l} [(\tilde{P}^{(l)}_{\mathrm{Re}})_{f,k} (\tilde{P}^{(l)}_{\mathrm{Im}})_{f,j} - (\tilde{P}^{(l)}_{\mathrm{Im}})_{f,k} (\tilde{P}^{(l)}_{\mathrm{Re}})_{f,j}]\} \tag{34}$$

and

$$[(\tilde{B}_{\mathrm{Im}})_{f,k}]^{(\mathrm{Im},f,k)} = \sum_{l=1}^{L} \{A_{k,l} A_{j,l} [(\tilde{P}^{(l)}_{\mathrm{Im}})_{f,k} (\tilde{P}^{(l)}_{\mathrm{Im}})_{f,j} + (\tilde{P}^{(l)}_{\mathrm{Re}})_{f,k} (\tilde{P}^{(l)}_{\mathrm{Re}})_{f,j}]\} \tag{35}$$

The right hand side of the equation $R^{(\mathrm{Im},f,k)}$ is constructed from the third terms of each of equations (26) and (27):

$$R^{(\mathrm{Im},f,k)} = \sum_{l} \{(\tilde{P}^{(l)}_{\mathrm{Re}})_{f,k} (\tilde{X}_{\mathrm{Im}})_{f,l} A_{k,l} - (\tilde{P}^{(l)}_{\mathrm{Im}})_{f,k} (\tilde{X}_{\mathrm{Re}})_{f,l} A_{k,l}\} \tag{36}$$

Thus, the overall linear equation associated with the derivative with respect to $(\tilde{B}_{\mathrm{Im}})_{f,k}$ is

$$\sum_{j=1}^{K} [(\tilde{B}_{\mathrm{Re}})_{f,j}]^{(\mathrm{Im},f,k)} (\tilde{B}_{\mathrm{Re}})_{f,j} + \sum_{j=1}^{K} [(\tilde{B}_{\mathrm{Im}})_{f,j}]^{(\mathrm{Im},f,k)} (\tilde{B}_{\mathrm{Im}})_{f,j} = R^{(\mathrm{Im},f,k)}. \tag{37}$$

Constructing F systems of $2K \times 2K$ equations Differentiating with respect to the real and imaginary components of each of the F Fourier coefficients for each of the K basis waveforms produces $F \times 2K$ linear equations in $F \times 2K$ variables. Careful inspection of equations (32) and (37) shows that a separate system of $2K \times 2K$ linear equations can be constructed for each frequency f (in other words, the overall linear system of equations is block-diagonal). Thus, the basis waveforms $\tilde{B}$ can be computed by solving F distinct linear systems, each with $2K$ variables and $2K$ equations.

3.2 Solving for basis waveforms with the Gaussian shape prior

The linear system of equations for determining basis waveform shapes described in Section 3.2.1 becomes under-determined or extremely ill-conditioned if at least one of basis waveforms is not observed for a particular cell (occurring, for instance, when the recorded cell lies near the edge of the electrode array). A Gaussian waveform shape prior that weakly constrains the learned waveform shapes to approximately resemble known waveform shapes is introduced to address this problem.

Let column vectors $\mathbf{b}_1, \mathbf{b}_2, ..., \mathbf{b}_K \in \mathbb{R}^T$ denote each of the compartment basis waveforms in time domain. The Gaussian prior for each of the basis waveforms has form

$$\mathbf{b}_k \sim \mathscr{N}(\mu_k, \Sigma_k) \tag{38}$$

where μ_i is the prior mean waveform of the specific compartment (e.g. a typical somatic waveform), and Σ is a temporal covariance matrix. The entries of temporal covariance matrix can be specified using a 1D Gaussian process kernel, for example, an exponentiated quadratic kernel $k(t_1, t_2) = \sigma^2 \exp\{-|t_1 - t_2|^2/(2l^2)\}$.

Like in Section 3.2.1, the waveform shape optimization with the Gaussian prior is performed in the Fourier domain, and thus the prior regularization penalty must be translated to the Fourier domain. Define F as the DFT analysis matrix for computing the one-dimensional Fourier transform of real-valued input. F is a complex-valued $(\lfloor \frac{T}{2} \rfloor + 1) \times T$ matrix, where T is the number of samples in time domain for the basis waveforms. We further define $F_{\text{Re}} \triangleq \text{Re}\{F\}$ and $F_{\text{Im}} \triangleq \text{Im}\{F\}$ as the real and imaginary components of the matrix F, respectively. F_{Re} is a real-valued $\lfloor \frac{T}{2} \rfloor + 1) \times T$ matrix, and F_{Im} is a real-valued $(\lfloor \frac{T-1}{2} \rfloor \times T$ matrix. Note that splitting F into real and imaginary components is only useful in the present case when the signal $\mathbf{b}$ is real-valued, since otherwise separating the real and imaginary components of the Fourier transform would not be linear operations.

Because the basis waveforms $\mathbf{b}$ are real-valued, the real component of the basis waveform Fourier transform is $\tilde{\mathbf{b}}_{\text{Re}} = F_{\text{Re}}\mathbf{b}$, and the imaginary component is $\tilde{\mathbf{b}}_{\text{Im}} = F_{\text{Im}}\mathbf{b}$. Define the real-valued vector $\mathbf{z} \triangleq [\tilde{\mathbf{b}}_{\text{Re}}^T \; \tilde{\mathbf{b}}_{\text{Im}}^T]^T$, and the $T \times T$ real-valued matrix G

$$G \triangleq \begin{bmatrix} F_{\text{Re}} \\ F_{\text{Im}} \end{bmatrix} \tag{39}$$

Because $\mathbf{z} = G\mathbf{b}$ is a linear transformation of the Gaussian random variable $\mathbf{b}$, $\mathbf{z}$ is also a jointly Gaussian random variable, and is distributed according to

$$\mathbf{z} \sim \mathscr{N}(G\mu, G\Sigma G^T) \tag{40}$$

which has pdf

$$p(\mathbf{z}) \propto \exp\{-\frac{1}{2}(\mathbf{z} - G\mu)^T (G_{\text{stack}}\Sigma G^T)^{-1}(\mathbf{z} - G\mu)\}. \tag{41}$$

The Gaussian prior penalty term associated basis waveform j, $\mathscr{P}_j$, is then rewritten as

$$\mathscr{P}_j \triangleq \frac{\lambda_p}{2}(\mathbf{z}_j - G\mu_j)^T (G\Sigma_k G^T)^{-1}(\mathbf{z}_j - G\mu_j) \tag{42}$$

and therefore the overall waveform shapes objective function, consisting of the sum of the mean square error terms and the Gaussian prior penalty terms, is

$$\sum_{l=1}^{L} \frac{1}{2}|(\tilde{B} \odot \tilde{P}_l)A_{:,l} - \tilde{X}_{:,l}|_2^2 + \frac{\lambda_p}{2}\sum_{j=1}^{K} \mathscr{P}_j. \tag{43}$$

3.2.1 Constructing linear equations from the first-order conditions

The linear system of equations from Section 3.2.1 must be updated to include the Gaussian prior penalty. Specifically, the contributions from the gradient of Gaussian prior penalty must be incorporated into the first-order conditions of optimality. The gradient of the Gaussian prior penalty with respect to the basis waveform Fourier coefficients $\mathbf{z}_k$ is

$$\boldsymbol{\nabla}_{\mathbf{z}_k}\left\{\frac{\lambda_p}{2}\sum_{j=1}^{K}\mathscr{P}_j\right\} = \lambda_p (G\Sigma_k G^T)^{-1}(\mathbf{z}_k - G\mu_k). \tag{44}$$

A notational difference regarding the indexing of the basis waveform Fourier coefficients must be resolved in order to incorporate the gradients in equation (44) with the first-order condition equations (32) and (32). Specifically, $(\tilde{B}_{\text{Re}})_{f,k} = \{\mathbf{z}_k\}_{(\text{Re},f)}$ and $(\tilde{B}_{\text{Im}})_{f,k} = \{\mathbf{z}_k\}_{(\text{Im},f)}$, where the

subscripts (Re, f) and (Im, f) represent the indices of the entries in $\mathbf{z}_k$ corresponding to the real and imaginary components of the Fourier coefficient at frequency f, respectively. Similarly, let $\{(G\Sigma_k G^T)^{-1}\}_{(\mathrm{Re},f),(\mathrm{Im},g)}$ denote the entry at the (Re, f) row and (Im, g) column of the matrix $(G\Sigma_k G^T)^{-1}$, e.g. the entry at the mth row and n^{th} column, where m is the index of the entry in $\mathbf{z}_k$ corresponding to the real component of the Fourier coefficient at frequency f, and n is the index of the entry in $\mathbf{z}_k$ corresponding to the imaginary component of the Fourier coefficient at frequency g.

First equation family, differentiating with respect to $(\tilde{B}_{\mathrm{Re}})_{f,k}$, a.k.a. $\{\mathbf{z}_k\}_{(\mathrm{Re},f)}$ Differentiating the Gaussian prior penalty with respect to $(\tilde{B}_{\mathrm{Re}})_{f,k}$, a.k.a. $(\mathbf{z}_k)_{(\mathrm{Re},f)}$, corresponds to the $(\mathrm{Re}, f)^{\mathrm{th}}$ entry of (44):

$$
\begin{aligned}
\frac{\partial}{\partial(\tilde{B}_{\mathrm{Re}})_{f,k}}\left\{\frac{\lambda_p}{2}\sum_{j=1}^{K}\mathscr{P}_j\right\} = \lambda_p \sum_g \{(G\Sigma_k G^T)^{-1}\}_{(\mathrm{Re},f),(\mathrm{Re},g)}(\tilde{B}_{\mathrm{Re}})_{g,k} \\
+\lambda_p \sum_g \{(G\Sigma_k G^T)^{-1}\}_{(\mathrm{Re},f),(\mathrm{Im},g)}(\tilde{B}_{\mathrm{Im}})_{g,k} \\
-\lambda_p \sum_g \{(G\Sigma_k G^T)^{-1}\}_{(\mathrm{Re},f),(\mathrm{Re},g)}\{G\mu_k\}_{(\mathrm{Re},f)} \\
-\lambda_p \sum_g \{(G\Sigma_k G^T)^{-1}\}_{(\mathrm{Re},f),(\mathrm{Im},g)}\{G\mu_k\}_{(\mathrm{Im},g)}
\end{aligned}
\tag{45}
$$

The first and second summations in equation (48) contain the variables (the entries $\tilde{B}$), while the third and fourth summations in (48) are constants. Defining

$$
\begin{aligned}
R_{\mathrm{prior}}^{(\mathrm{Re},f,k)} \triangleq \lambda_p \sum_g \{(G\Sigma_k G^T)^{-1}\}_{(\mathrm{Re},f),(\mathrm{Re},g)}\{G\mu_k\}_{(\mathrm{Re},f)} \\
+\lambda_p \sum_g \{(G\Sigma_k G^T)^{-1}\}_{(\mathrm{Re},f),(\mathrm{Im},g)}\{G\mu_k\}_{(\mathrm{Im},g)},
\end{aligned}
\tag{46}
$$

inclusion of the Gaussian prior into equation (32) results in

$$
\begin{aligned}
\sum_{j=1}^{K}[(\tilde{B}_{\mathrm{Re}})_{f,j}]^{(\mathrm{Re},f,k)}(\tilde{B}_{\mathrm{Re}})_{f,j} + \sum_{j=1}^{K}[(\tilde{B}_{\mathrm{Im}})_{f,j}]^{(\mathrm{Re},f,k)}(\tilde{B}_{\mathrm{Im}})_{f,j} \\
+\lambda_p \sum_g \{(G\Sigma_k G^T)^{-1}\}_{(\mathrm{Re},f),(\mathrm{Re},g)}(\tilde{B}_{\mathrm{Re}})_{g,k} \\
+\lambda_p \sum_g \{(G\Sigma_k G^T)^{-1}\}_{(\mathrm{Re},f),(\mathrm{Im},g)}(\tilde{B}_{\mathrm{Im}})_{g,k} = R^{(\mathrm{Re},f,k)} + R_{\mathrm{prior}}^{(\mathrm{Re},f,k)}.
\end{aligned}
\tag{47}
$$

Second equation family, differentiating with respect to $(\tilde{B}_{\mathrm{Im}})_{f,k}$, a.k.a. $\{\mathbf{z}_k\}_{(\mathrm{Im},f)}$ Differentiating the Gaussian prior penalty with respect to $(\tilde{B}_{\mathrm{Im}})_{f,k}$, a.k.a. $\{\mathbf{z}_k\}_{(\mathrm{Im},f)}$ corresponds to the

$(\mathrm{Im}, f)^{\mathrm{th}}$ entry of (44):

$$
\begin{aligned}
\frac{\partial}{\partial(\tilde{B}_{\mathrm{Im}})_{f,k}}\left\{\frac{\lambda_p}{2}\sum_{j=1}^{K}\mathscr{P}_j\right\} = \lambda_p \sum_g \{(G\Sigma_k G^T)^{-1}\}_{(\mathrm{Im},f),(\mathrm{Re},g)}(\tilde{B}_{\mathrm{Re}})_{g,k} \\
+\lambda_p \sum_g \{(G\Sigma_k G^T)^{-1}\}_{(\mathrm{Im},f),(\mathrm{Im},g)}(\tilde{B}_{\mathrm{Im}})_{g,k} \\
-\lambda_p \sum_g \{(G\Sigma_k G^T)^{-1}\}_{(\mathrm{Im},f),(\mathrm{Re},g)}\{G\mu_k\}_{(\mathrm{Re},f)} \\
-\lambda_p \sum_g \{(G\Sigma_k G^T)^{-1}\}_{(\mathrm{Im},f),(\mathrm{Im},g)}\{G\mu_k\}_{(\mathrm{Im},g)}
\end{aligned}
\tag{48}
$$

Defining

$$
\begin{aligned}
R_{\mathrm{prior}}^{(\mathrm{Im},f,k)} \triangleq \lambda_p \sum_g \{(G\Sigma_k G^T)^{-1}\}_{(\mathrm{Im},f),(\mathrm{Re},g)}\{G\mu_k\}_{(\mathrm{Re},f)} \\
+\lambda_p \sum_g \{(G\Sigma_k G^T)^{-1}\}_{(\mathrm{Im},f),(\mathrm{Im},g)}\{G\mu_k\}_{(\mathrm{Im},g)},
\end{aligned}
\tag{49}
$$

and thus inclusion of the Gaussian prior into (37) results in

$$
\begin{aligned}
\sum_{j=1}^{K}[(\tilde{B}_{\mathrm{Re}})_{f,j}]^{(\mathrm{Im},f,k)}(\tilde{B}_{\mathrm{Re}})_{f,j} + \sum_{j=1}^{K}[(\tilde{B}_{\mathrm{Im}})_{f,j}]^{(\mathrm{Im},f,k)}(\tilde{B}_{\mathrm{Im}})_{f,j} \\
+\lambda_p \sum_g \{(G\Sigma_k G^T)^{-1}\}_{(\mathrm{Im},f),(\mathrm{Re},g)}(\tilde{B}_{\mathrm{Re}})_{g,k} \\
+\lambda_p \sum_g \{(G\Sigma_k G^T)^{-1}\}_{(\mathrm{Im},f),(\mathrm{Im},g)}(\tilde{B}_{\mathrm{Im}})_{g,k} = R^{(\mathrm{Im},f,k)} + R_{\mathrm{prior}}^{(\mathrm{Im},f,k)}.
\end{aligned}
\tag{50}
$$

Constructing a single $KN \times KN$ system of equations The Gaussian prior penalty couples coefficients of all frequencies for the same basis waveform together, and thus the block diagonal structure from Section is lost. Solution of the first-order conditions with the Gaussian prior penalty therefore requires solving a single $KN \times KN$ system of linear equations.

References

[1] A. Beck and M. Teboulle, "A Fast Iterative Shrinkage-Thresholding Algorithm for Linear Inverse Problems," en, *SIAM Journal on Imaging Sciences*, vol. 2, no. 1, pp. 183–202, Jan. 2009, ISSN: 1936-4954. DOI: 10.1137/080716542. [Online]. Available: http://epubs.siam.org/doi/10.1137/080716542 (visited on 05/05/2022).

[2] J. Liu, S. Ji, and J. Ye, "Multi-Task Feature Learning Via Efficient 2,1-Norm Minimization," en, in *Proceedings of the Twenty-Fifth Conference on Uncertainty in Artificial Intelligence*, Montreal, Canada: AUAI Press, 2009, p. 10.
